# Nuclear domain 10-associated proteins recognize and segregate intranuclear DNA/protein complexes to negate gene expression

**DOI:** 10.1186/1743-422X-9-222

**Published:** 2012-09-28

**Authors:** Yisel A Rivera-Molina, Bruno R Rojas, Qiyi Tang

**Affiliations:** 1 Department of Microbiology/RCMI Program, Ponce School of Medicine and Health Sciences, Ponce, 00716, Puerto Rico

**Keywords:** Nuclear domain 10 (ND10), Protein-DNA complexes, Amplicon, Lac operator, Lac repressor, PML, Daxx, Transcript location, Nuclear bodies

## Abstract

**Background:**

DNA viruses, such as herpes simplex virus type 1 (HSV-1), Simian virus 40 (SV40), and Cytomegaloviruses (CMV), start their replicative processes and transcription at specific nuclear domains known as ND10 (nuclear domain 10, also called PML bodies). It has been previously determined that for HSV-1 and SV40, a short DNA sequence and its binding protein are required and sufficient for cell localization of viral DNA replication and gene transcription.

**Results:**

Our recent observations provide evidence that a foreign (not endogenous) DNA/protein complex in the nucleus recruits ND10 proteins. First, the complexes formed from the bacterial lac operator DNA and its binding protein (lac repressor), or from HPV11 (human papillomavirus 11) origin DNA and its binding protein (E2), co-localized with different ND10 proteins. Second, the HSV-1 amplicon without inserted lac operator DNA repeats distributed in the nucleus randomly, whereas the amplicon with lac operator DNA repeats associated with ND10, suggesting that DNA-binding proteins are required to localize at ND10. The cellular intrinsic DNA/protein complex (as detected for U2 DNA) showed no association with ND10. Furthermore, our examination of PML−/−, Daxx−/−, and Sp100-negative cells led to our discovering that DNA/protein complexes recruit ND10 protein independently. Using the GFP-LacI/Operator system, we were able to direct the transfected DNA to ND10 and found that gene expression was significantly repressed when the transfected DNA was directed to ND10.

**Conclusion:**

Taken together, the results suggest that cells recognize DNA/protein complexes through a mechanism that involves interaction with the ND10-associated proteins.

## Introduction

Mammalian cells contain differentially functional compartments called organelles that are separated from cytoplasm by a lipid bilayer membrane. The nucleus is an extremely dynamic organelle and highly organized compartment with multiple functions [reviewed in [1-3]]. When analyzed by indirect immunofluorescence microscopy, many nuclear proteins are seen to localize in distinct structures with punctate staining patterns [4,5]. Nuclear structures, such as speckles, paraspeckles, nucleoli, Cajal bodies, polycomb bodies, and nuclear domain 10 (ND10, a.k.a. promyelocytic leukemia— PML body) are formed primarily by protein-protein, protein-RNA, or protein-DNA interactions [6]. ND10 is a subnuclear structure that gathers many different SUMOylated nuclear proteins (such as Daxx and SP100). The formation of ND10 depends on PML protein. Past observations confirm that PML knockout cells lack ND10 and that transfecting exogenous PML into PML knockout cells results in the restoration of ND10. Most DNA viruses replicate DNA and transcribe genes in the nucleus after their genomic DNA enters the nucleus by facilitated transport through the nuclear pore complex [7]. Once inside the nucleus, viral genomes distribute randomly, but it appears that only those at ND10 replicate and transcribe predominantly [8-11], suggesting specifically that the environment at ND10 is particularly advantageous for the virus. However, the ND10 proteins (such as PML, Sp100, and Daxx) are interferon-upregulated and have repressive effects on viral replication [12-23]. Moreover, most DNA viruses encode an immediate-early protein that induces the dispersion of ND10 [8,24-27], and in the absence of these viral proteins, replication is severely retarded [11,27,28]. These findings have led to the hypothesis that ND10 sites are also a part of a nuclear defense mechanism [29]. Therefore, the effects of ND10 on viral replication remain to be settled.

What is known definitively is that RNA transcripts distribute throughout the nucleus in either a diffuse or speckled pattern [10]. As determined for both SV40 and HSV-1, the origin DNA was necessary but not sufficient for the virus to transcribe RNA and replicate DNA at ND10 [10,30]. The origin-binding proteins (large T-antigens of SV40 or ICP4 of HSV-1) were also required, suggesting that a DNA-protein complex precedes virus-ND10 association [10,30]. The Everett group has observed that the HSV-1 genome is associated with ND10 proteins after infection and demonstrated that this association was formed by a new aggregation of ND10 components rather than by the migration of preexisting intact ND10 structures [31]. Furthermore, Everett and colleagues recently discovered that the SUMO pathway is important for the recruitment of ND10-associated proteins to the HSV-1 genome [32]. The studies of both SV40 and HSV-1 led to a new concept, that DNA-protein complexes might be sensed by ND10 or ND10 proteins. In *in vivo* studies observing chromatin structure, the Belmont group introduced repetitive lac operator (lacO) sequences and a tightly binding lac repressor protein that was fused with GFP (GFP-lac repressor) into the nucleus and found that the GFP-lac repressor/Operator complexes localize at ND10 [33-35]. Later, the Spector group observed a dynamic interaction between PML and GFP-lac repressor/Operator complexes [36]. Those observations led to our conceptual hypothesis that ND10 might be a “sensor” for recognizing DNA/protein complexes [37]. We wondered whether the GFP-lac repressor/Operator system can be used to determine the effects of ND10 on gene expression.

In the present study, we first showed that the lac operator alone is not associated with ND10, although ND10 recognizes GFP-lac repressor/Operator complexes at a rate of 100%. We next inserted repetitive DNA into HSV-1 amplicons to make them visible and useful in the analysis of viral DNA sequences that, in the presence of DNA binding protein, are deposited at ND10. Our findings with respect to infectious DNA and transfected DNA suggest that instead of having sensors for proteins, cells possess mechanisms for recognizing deposits of DNA/protein complexes and segregating such complexes into loci containing several ND10-associated proteins. In addition, we found that HPV origin DNA/Origin-binding protein (E2) complexes can also be recognized by ND10. However, endogenous DNA/protein complexes were not associated with ND10. Therefore, our observations suggest that foreign DNA/protein complexes might be able to recruit or be recognized by ND10 proteins. Most importantly, the gene expression at ND10 was detected with less intensity than that was not associated with ND10, which demonstrated that ND10 is a restrictive site for gene expression for the tested DNA/protein complexes.

## Results

### A DNA/protein complex derived from bacteria recruits ND10-associated proteins

**Figure 1:**
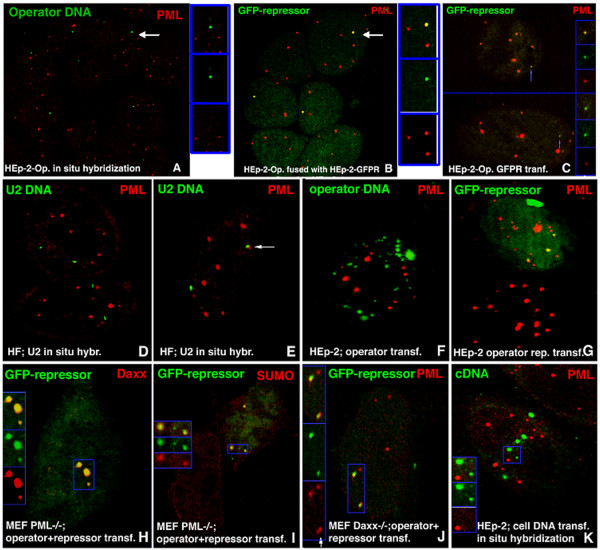
**Localization of integrated and transfected operator repeats containing no transcription units.** Components labeled are indicated in the upper corners of the panels with their respective colors. (**A**) HEp-2-Op (derived from HEp-2 cells integrated with 10 kb = 256 operator repeats) probed by *in situ* hybridization for the location of the operator repeat insert; the operator DNA does not colocalize with PML. (**B**) HEp-2-Op (256 repeats) cells fused with HEp-2gfplacI cells. The single integrated operator repeat insert from 3 HEp-2-Op cell nuclei is found within PML-stained regions (yellow). The GFP-repressor is diffusely distributed in the three HEp-2gfplacI cell nuclei. (**C**) HEp-2-Op cells with 128 operator repeats were transfected with the GFP-repressor. Integrated operator repeats appear green (arrow). Insets show the color-separated small operator repeats at higher magnification. (**D**) HF cells probed by *in situ* hybridization for the location of the U2 gene locus relative to PML. The respective loci do not colocalize. (**E**) The same as (**D**), but showing a U2 locus localized adjacent to PML (arrow). (**F**) HEp-2 cells transfected with operator repeats and probed by *in situ* hybridization for operator location relative to PML. Operator plasmid aggregates do not colocalize with PML. (**G**) HEp-2 cells cotransfected with operator repeats and the GFP-repressor expression vector. The “green” operator/repressor complexes in the nucleus colocalize with PML. (**H**) MEF PML−/− cotransfected with operator repeats and the GFP-lac repressor expression vector and labeled for Daxx. Daxx colocalizes with the operator/repressor complex. The inset shows the area framed in blue at a higher magnification and with the colors separated. (**I**) MEF PML−/− cotransfected with operator repeats and the GFP-repressor expression vector and labeled for SUMO, which colocalizes with the operator/repressor complex. The inset shows the area framed in blue at a higher magnification and with the colors separated. (**J**) Mouse Daxx−/− cells cotransfected with operator repeats and the GFP-repressor expression vector and labeled for PML, which colocalizes with the operator/repressor complex. The inset shows the area framed in blue at a higher magnification and with the colors separated. (**K**) HEp-2 cells transfected with a plasmid-containing cellular heat shock promoter. *In situ* hybridization shows plasmid aggregates not colocalizing with PML. The inset shows the area framed in blue at a higher magnification and with the colors separated.

The essential components resulting in viral transcription at ND10 have been identified by our previous studies of HSV-1 and SV40 (58, 59). The requirements can be generalized as a specific viral DNA sequence (the origin), a transcription unit, and a viral protein that binds to the origin DNA, presumably as a DNA/protein complex. Linear integrated arrays of transcription units each flanked by the bacterial operator/repressor have been found in association with PML (64). We asked ourselves whether the requirement for a DNA fragment might be reduced to just the protein binding sequence. We constructed plasmids containing bacterial lacO (operator) repeats that specifically bind to the lac repressor without viral origins and without eukaryotic transcription units (see Materials and Methods). Transfected HEp-2 cell lines were selected that had various numbers of integrated lacO repeats resulting in small integration sites (HEp-2-Op with 128 repeats cells) or larger sites comparable to the size of an average ND10 (HEp-2-Op with 256 repeats cells), as determined by *in situ* hybridization. As determined previously by time-lapse analysis with GFP-labeled PML, ND10 sites are essentially immobile (45), with the exception of occasional, very small PML-containing aggregates (42). *In situ* hybridization of cells with the operator sequences showed that most (74%) integrated operator repeats did not associate with ND10 (Figure 1A), as had previously been shown for the operator-flanked transcription units (64). However, about 26% of all operator sequences were found to be adjacent to or to partially overlap ND10 (Table 1).

**Table 1:** **Quantitative assessment of thepositions of integrated operatorsequences and control genomicsites relative to PMLor Sp100**

**DNA locus**	**Cell line**	**Colocali-zation**	**Side by side orpartial overlap**	**No association**	**Number of nuclei evaluated**
large operator insert w/o repressor	QT10 HEp-2-Op256	0%	26%	74%	1000
large operator insert + GFP-repressor	QT10 HEp-2-Op256	100%	0%	0%	1000
small operator insert w/o repressor	QT5 HEp-2-Op128	0%	12%	87%	1000
small operator insert + GFP-repressor	QT5 HEp-2-Op128	72%	0%	28%	1000
U2	human fibroblasts	0%	17%	83%	960
D1Z2	human fibroblasts	0%	11%	89%	1280
β-actin	human fibroblasts	0%	22%	78%	940
collagen Iα1	human fibroblasts	0%	24%	76%	1070

Association of ND10 with integrated repetitive sequences (*lac* operator, with or without binding GFP-repressor), endogenous repetitive sequences (U2, D1Z2), and endogenous non-repetitive genes (β-actin, collagen Iα1). DNA loci were visualized either by FISH or via the binding GFP-repressor, and cells were simultaneously stained for PML or Sp100 to assess the percentage of loci that associate with these proteins (side-by-side and/or overlapping). Loci were counted as *colocalizing* if the site was totally covered by PML or Sp100, as *side-by-side* if touching or partially overlapping, and as *not associated* if a clear space could be discerned between the two structures. Genomic sites consisting of repetitive DNA (U2, D1Z2) do not show a higher association rate with ND10 compared to that seen with single copy genes (β-actin, collagen Iα1), and neither one colocalizes with PML or Sp100 (QT10: HEp-2-Op256; QT5: HEp-2-Op128).

To test whether integrated operator repeats without the transcription unit would recruit ND10-associated proteins, we supplied HEp-2-Op cells with the GFP-lac repressor from cells producing this protein. For that purpose, we constructed a HEp-2 cell line that produced small amounts of the GFP-lac repressor (HEp-2gfplacI), which was distributed diffusely. When HEp-2gfplacI cells were fused with HEp-2-Op (256 repeats) cells, all operator integration sites, recognized as a single green operator/GFP-lac repressor complex per nucleus, also became PML positive (Figure 1B). The same results were observed when the GFP-lacI was provided by transfection of gfp-lacI-expressing plasmid (Figure 1C). Staining for the other ND10-associated proteins tested yielded the same results (see Table 2). When the HEp-2-Op cell line containing the smaller inserts was fused with HEp-2gfplacI cells or transfected with the GFP-lac repressor plasmid, not all integrated operator/repressor complexes were recognizably positive for PML (Table 1). In HEp-2-Op cells, we have never observed the small green signal of the operator/repressor complex within a large accumulation of PML, suggesting that the DNA/protein complex does not nucleate the formation of ND10. Together, these findings provide clear evidence that the accumulation of the proteins within the operator-defined domain is induced by the binding of the repressor to the integrated operator repeats and that no transcription unit other than the operator sequence is required.

**Table 2:** **ND10 proteins found inassociation with transfected orintegrated operator**

**Cell type**	**Operator/repressor**	**PML**	**SUMO-1**	**Daxx**	**Sp100**	**NDP55**	**HP1**
HEp-2	cotransfected	+	+	+	+	+	-
HEp-2	only operator transfected (FISH)	-	-	-	-	-	-
MEF PML−/−	cotransfected	0	+	+	+	+/−	-
MEF Daxx−/−	cotransfected	+	+	0	+	+	-
293 Sp100 -	cotransfected	+	+	+	0	+	-
HEp-2-Op256	operator integrated, repressor transfected	+	+	+	+	+	-

Various cell lines, some missing specific ND10-associated proteins (0), were co-transfected with plasmids carrying either the *lac* operator repeats or the GFP-repressor protein and evaluated by fluorescent microscopy to determine whether specified ND10-associated proteins colocalized with (+) or were present apart from the operator GFP-repressor complex (−). For a quantitative assessment of various integrated operator sequences, see also Table 1. Repeats in the Presence or Absence of GFP-repressor Protein.

Since the integrated operator repeats represent an array of the same sequence, we asked ourselves whether arrays of cellular DNA sequences associated with cellular proteins might also accumulate enough ND10-associated proteins and thus generate ND10. Two sequences were compared: the transcribed tandem array U2 gene cluster [38] and the non-coding mid-satellite repetitive sequences D1Z2 [39]. As additional controls, two single copy genes, actin and collagen Iα1, were selected because they are located at the surface of chromosomes that are facing the interchromosomal space [39-41] where ND10 sites are located [40]. Staining for PML was followed by FISH analysis on exponentially growing human fibroblasts, and the location of DNA loci relative to ND10 was assessed. Most DNA hybridization foci of the 4 genes were not associated with ND10 (shown for U2 in Figure 1D). However, some were found to be overlapping or juxtaposed to ND10 (Figure 1E). As summarized in Table 1, we quantitated the association rate of ND10 with protein/DNA complexes (endogenous or exogenous) by counting around one thousand nuclei. Loci consisting of endogenous repeat unit DNA (U2, D1Z2) do not show a higher association rate with ND10 compared to the single-copy genes β-actin and collagen Iα1 (Table 1); they were in fact found beside ND10 at a lower frequency (11% for D1Z2 and 17% for U2). Since these genes are located on the surfaces of chromosomes [38,41], and ND10 are located in the interchromosomal space [40], the exclusion of ND10 from chromosomes and nucleoli may place these genes in the observed frequency of opposition, despite random association. More importantly, however, the repetitive cellular DNA tested does not colocalize with ND10-associated proteins, as compared with the integrated DNA. Integrated DNA alone is not related to ND10 (the colocalization rate is 0%); however, if the integrated DNA was bound to foreign protein, the colocalization with ND10 is obvious (100% for large operator insert and 72% for small operator insert).

### ND10 proteins are independently associated with the operator/repressor complex

In light of the apparent similarity between the presence of ND10-associated proteins in operator/repressor complexes and in viral DNA/protein complexes, we attempted to determine whether the lack of the essential ND10-inducing protein, PML, might affect the recruitment of other individual ND10-associated proteins such as Daxx and Sp100. We transfected HEp-2 cells with the same plasmids used for integration into HEp-2-Op cells. *In situ* hybridization of cells transfected with these plasmids showed them as large aggregates in the nucleus. The GFP-lac repressor alone was homogeneously dispersed throughout the nucleus, as was the case in the constitutively expressing cells (similar to the distribution in Figure 1B; lower two nuclei). The operator-containing plasmid alone was present as clumps in the space surrounding the nucleus and in variably sized aggregates inside the nucleus but not at ND10 sites (Figure 1F). After the cotransfection of operator repeats and gfplacI-expressing plasmids, a direct visualization of operator/repressor complexes showed that these complexes were associated with PML (Figure 1G). The operator/repressor complexes in the nucleus were not of uniform size and appeared to consist of multiple plasmids. Most colocalized with PML, indicating that a foreign DNA/protein complex introduced into the nucleus is either deposited at ND10 or recruits ND10-associated proteins. The large plasmid aggregates enter the nucleus, so they can move, although they might be restricted in their mobility as shown for anti-sense RNA aggregates [42].

The formation of a complex by operator sequences and the GFP-lac repressor by cotransfection provided an assay to determine whether the absence of ND10 as a structure (as occurs in PML−/− cells) would prevent the DNA/protein complex-based recruitment of other ND10-associated proteins and with short tandem transgene repeats [43]. Cell lines, previously determined to be deficient in certain ND10-associated proteins, were cotransfected with the operator repeats and repressor plasmids and tested with specific antibodies against ND10-associated proteins in order to determine whether they colocalized with the operator/repressor. In HEp-2 cells, all of the ND10-associated proteins tested (except HP1) were found together with the operator/repressor complexes but not with the transfected cellular DNA (Table 2). In the absence of the repressor, none of the operator repeats were surrounded by any of the ND10-associated proteins (Table 2). Most interesting are the results from PML−/− cells, where all other ND10-associated proteins are dispersed and ND10 as such do not exist [44]. In MEF PML−/− cells, we found Daxx colocalized with the operator/repressor complex (Figure 1H), indicating that this protein is recruited to the complex directly, and not through recruitment by PML. A SUMO signal was detected (by anti-SUMO-1 antibody) with the operator/repressor complex in the PML−/− cells (Figure 1I). Antibodies to NDP55, an as yet uncharacterized ND10-associated protein, were also present, but only in minor accumulations (not shown). This set of tests shows that PML, apparently essential for ND10 formation, is not essential for recruitment of other ND10-associated proteins to a foreign DNA/protein complex. Thus, these proteins cannot be considered ND10 or PML bodies.

Since Daxx reportedly binds directly or indirectly to a specific DNA sequence [45], we attempted to determine whether Daxx is required for the association of the operator-repressor complex with other ND10-associated proteins. We used Daxx knock-out cells derived from Daxx knock-out mouse embryos [46]. After cotransfection of these cells, sites of operator/repressor complexes were found adjacent to various ND10 proteins (Table 2, and shown for PML in Figure 1J). The image is interpreted as showing that an operator/protein complex derived from transfected DNA was deposited beside ND10 and had recruited PML into its domain. Control transfections with cellular DNA showed no accumulation of ND10-associated proteins with either the U2 containing plasmid or plasmids containing the Hsp70 promoter, whether or not heat shock was applied (shown for the heat shock factor-containing plasmid in Figure 1K).

All tested ND10 proteins were found together with the operator/repressor complex in Sp100-negative 293 cells except HP-1(Table 2). Sp100 is therefore also not essential for the recruitment of ND10 proteins. Analysis of the cell lines missing PML, Daxx, or Sp100 showed that the structural integrity of ND10 is not necessary for the attraction of various ND10-associated proteins by operator/repressor sequence accumulations. Instead, the proteins are recruited to the operator/repressor complex from the nucleoplasm or ND10, and Daxx, Sp100, and PML are all unnecessary for the recruitment of other proteins into the domain occupied by the operator/repressor complex.

### Another foreign DNA/protein complex derived from HPV11 recruits ND10-associated proteins

**Figure 2:**
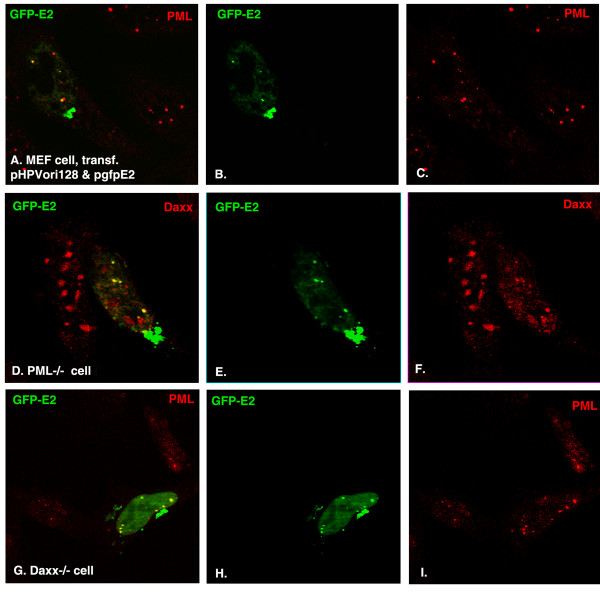
**Localization of HPV origin DNA repeats in the nucleus after cotransfection with GFP-E2–expressing plasmid.** Components labeled are indicated in the upper corners of the panels with their respective colors. (**A**)-(**C**): MEF cells cotransfected with pHPVori128 and pgfpE2; the oriDNA-GFPE2 complex is shown in green (**B**) and PML is shown in red (**C**); the merged picture is in (**A**). (**D**-**F**): The same as in (**A**-**C**), but cells are MEF PML−/− cells and show Daxx in red (**F**). (**G**-**I**), the same as in (**A**-**C**), but the cells are MEF Daxx−/− cells.

We and other groups have shown that several viral origin/protein complexes (such as HSV-1 and SV40) colocalize with ND10 [10,30]; here, we tested whether the HPV origin/origin-binding protein complex could recruit ND10 proteins. We constructed a plasmid that contained 128 copies of the core origin of HPV11 (pHPVori128) and transfected it together with a gfpE2-expressing plasmid (pgfpE2, containing GFP-fused E2 origin binding protein) into PML+/+ (Figure 2A-C), PML−/− (Figure 2D-F), and Daxx−/− cells (Figure 2G-I). Twenty-four hours later, the cells were fixed, permeabilized, and immunostained so that we could visualize the localization of E2 and ND10-associated proteins. As can be seen in Figure 2, after co-transfection of the two plasmids, origin DNA and its binding protein formed complexes in the nucleus (there are also clumps formed outside of the nucleus), and these complexes colocalized with the ND10-associated proteins PML and Daxx. Similar to that observed for operator/repressor complexes shown in Figure 1, the colocalization of DNA/protein complexes with Daxx is independent of PML (Figure 2D-F), and colocalization with PML is also independent of Daxx (Figure 2G-I). We quantitated the association rate by counting one hundred positively cotransfected cells, and the colocalization rate was at 75% for MEF cells, 78% for PML−/− cells, and 80% for Daxx−/− cells. That is to say, we have found another foreign DNA/protein complex that recruits ND10-associated proteins. 

### ND10 protein in the foreign DNA/protein complex can be dispersed by IE1

**Figure 3:**
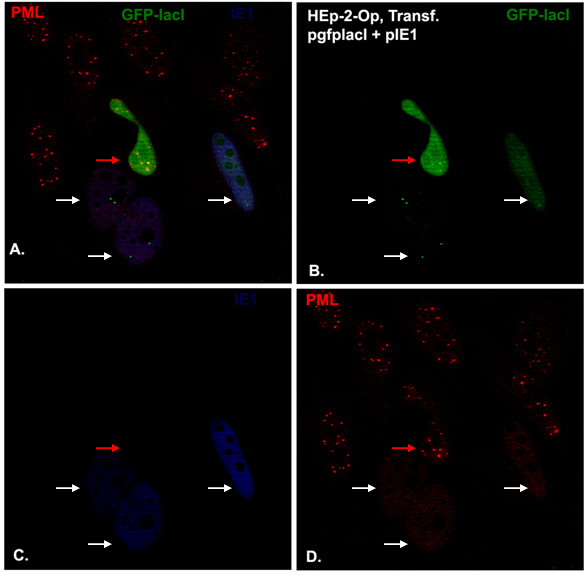
**Localization of integrated operator repeats containing no transcription units after cotransfection with gfplacI- and CMV IE1-expressing plasmids.** Components labeled are indicated in the upper corners of the panels with their respective colors. HEp-2-Op cells were cotransfected with pgfplacI and pIE1 for 24 hours, and then the cells were stained blue for IE1 (**C**) and red for PML (**D**). The lac Operator/GFP-lac repressor complexes are shown in green (**B**). All colors are merged in (**A**).

One of the properties of ND10 is that it can be dispersed by several DNA viral proteins, such as ICP0 of HSV-1 and IE1 of cytomegalovirus [11,25,47,48]. We asked whether the ND10 formed with foreign DNA/protein complexes possesses this same characteristic. To answer this question, we cotransfected the lac repressor and pIE1 (HCMV IE1-expressing plasmid) into HEp-2-Op cells for 24 hours. After being fixed and permeabilized, the cells were immunostained (Figure 3). As can be seen, green DNA/protein complexes (one or two dots in each cell) were formed in four cells as shown by arrows. Among them, three cells (shown by white arrows) expressed IE1 (blue) and ND10 were dispersed by IE1. In the cell (shown by red arrow in the Figure 3A, B, and D) that was positive for GFP-lac repressor and negative for IE1, ND10 colocalized with the foreign DNA/protein complex. Therefore, ND10 in the foreign DNA/ protein complex can also be dispersed by viral proteins. 

### The foreign DNA/protein complex guides RNA transcription at ND10

**Figure 4:**
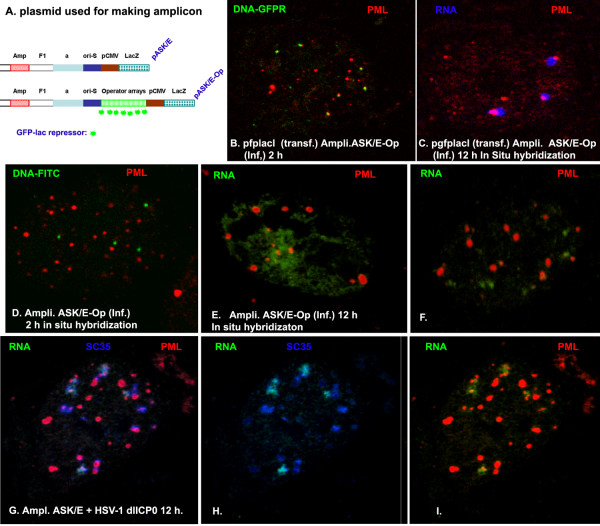
**Gene transcription, ND10, and splicing compartments. (A)** The plasmids used for making HSV-1 amplicons. (**B**) HEp-2 cells were transfected with pgfplacI; 6 hours later, the cells were super-infected with the amplicon ASK/E-Op for 2 hours. Then the cells were stained for ND10 using PML antibody and Texas Red-conjugated secondary antibody. Pictures were taken to show the relationship of ND10 and input amplicon DNA. (**C**) The same as (**B**), but the infection with the amplicon was at 12 hours, and the *in situ* hybridization experiment examined RNA (in blue), showing the relationship between lacZ gene transcription and ND10. (**D**) HEp-2 cells were infected with the amplicon (ASK/E-Op) for 2 hours; *in situ* hybridization was performed to show amplicon DNA (green). Immunofluorescence shows ND10 (red). (**E**) The same as (**D**), but the infection was for 12 hours; *in situ* hybridization was performed to show diffused β-gal RNA (DNA was degraded by DNAse treatment). (**F**) The same as (**E**), but the picture was taken to show the putative RNA. (**G**) HEp-2 cells were infected with the amplicon (ASK/E) and ICP0-deleted HSV-1 (as helper virus) for 12 hours; FISH was performed to show the distribution of lacZ RNA (green), ND10 (red), and splicing compartments (blue). (**H**) The same as (**G**) to show the relationship of RNA and splicing compartments. (**I**) The same as (**G**), to show the relationship of ND10 and RNA.

DNA viruses replicate DNA and transcribe RNA, predominantly at ND10 [10]. The predominance of RNA accumulation at ND10 can be explained by the fact that viral DNA docks at ND10 at the time of infection. The procedure appears to be dependent on the formation of DNA/protein complexes, as was determined for SV40 and HSV-1 [10,30]. If that is the case, we wonder whether we can facilitate gene expression at ND10 by guiding transfected or infected DNA to ND10. To confirm our assumption that foreign DNA/protein complexes can recruit ND10 proteins, we introduced lac operator DNA repeats (10 kb = 256 arrays) into an HSV-1 amplicon, which resulted in pASK/E-Op (Figure 4A). To determine whether ND10 protein could be recruited to amplicon DNA or DNA/protein complexes, we infected the amplicon pASK/E-Op into HEp-2 cells that had been transfected with pgfplacI for 6 hours. Two hours later, the cells were stained for PML; as can be seen in Figure 4B, the infected amplicon DNA genomes colocalized with or overlapped ND10 (shown by PML in red). As a control, we infected the amplicon pASK/E-Op alone into HEp-2 cells and performed *in situ* hybridization for DNA using the probe that is specific to the amplicon plasmid. As can be seen in Figure 4D, the infected amplicon DNA does not associate with ND10, as expected. Therefore, the GFP-lac repressor/operator DNA complexes are required for the DNA to associate with ND10. 

The only gene carried by the amplicon is β-gal. We wondered whether RNA transcription of β-gal is retained at ND10 with the amplicon. We infected amplicon ASK/E-Op into HEp-2 cells that had been transfected with pgfplaci for 6 hours. Then we performed *in situ* hybridization for β-gal RNA (DNA was degraded by DNaseI before hybridization) in blue. As can be seen in Figure 4C, the RNA was side by side with ND10 (red). In this system, RNA was detected as punctate speckles in 100% of the cells that were positively infected (we counted 100 cells). Without pgfplacI, the RNA distributed diffusely in about 80% of the nuclei (Figure 4E) and did so in a punctate manner in 20% of the nuclei (100 cells were counted) that were not associated with ND10 (Figure 4F). Therefore, the GFP-lac repressor/operator system can be used to facilitate the input DNA to ND10, which will render gene transcription at ND10.

We wanted to find out whether the β-gal RNA retained at ND10 is associated with splicing compartments. To that end, we infected amplicon ASK/E into HEp-2 together with a helper virus (HSV-1 dlICP0). The amplicon DNA and HSV-1 proteins (ICP8, ICP4) can form foreign DNA-protein complexes and their transcription is at ND10 [10]. As we expected, the RNA transcription occurred at ND10 (Figure 4I), and the transcribed lacZ RNA was also retained in splicing compartments (shown by SC35 in blue, Figure 4H). The colors were merged in Figure 4G to show the relationships between ND10, the splicing compartments, and RNA.

### Transfected gene transcription was inhibited at ND10

**Figure 5:**
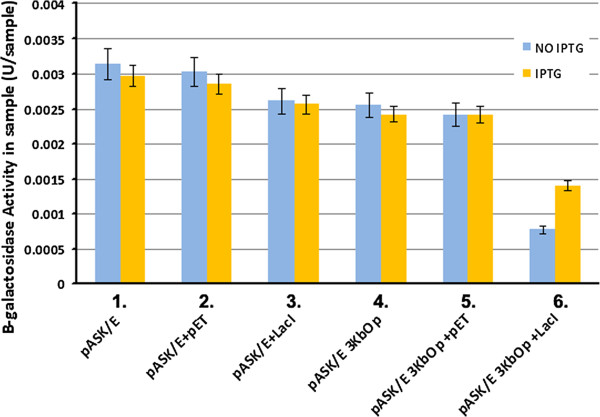
**Beta-galactosidase assay.** Detection of the activity of β-galactosidase was carried out using the β-Galactosidase Reporter Gene Staining Kit from Sigma-Aldrich, following the manufacturer’s protocol. Twenty-four hours after transfection of the plasmids (as indicated in Figure 5) in 293-T cells, the cells were washed three times with PBS and lysed with lysis buffer. Mock-infected 293T cells were used as controls. The samples were normalized to the sample amount of total protein. The β-Galactosidase activity in the sample was calculated taking into consideration the OD, final reaction volume, the absorbance of 1mM for an optical path of 1cm, and the incubation period (t_min_). For the assays involving isopropyl-thio-b-D-galactopyranoside (IPTG), a final concentration of 0.4mM of IPTG was used. IPTG was added to the supplemented medium and left overnight. Each experiment was performed in triplicate and an average value obtained.

As a nuclear structure, ND10’s effects on gene expression have not yet been determined. Based on the observations from the present studies that foreign DNA and its binding protein are essential and sufficient for the foreign DNA to colocalize with ND10, we hypothesized that ND10’s function can be examined using the lacO/GFP-lac repressor system to facilitate gene transcription at ND10 or not at ND10. We cotransfected pASK/E or pASK/E3kbOp (carrying 3 kb operator repeats and a lac Z gene under the control of a CMV promoter) with pET (GFP alone) or pgfplacI (producing GFP-lacI fusion protein) into 293-T cells and performed a β-galactose assay. Since only the lacO/GFPlacI complex can overlap with ND10, the cotransfection of pASK/E3kbOp with pET was used as a control for the gene expression outside of ND10. As can be seen, no significant differences were observed for the gene expression between the cotransfection of pASK/E with pET and pgfplacI. Comparing the cotransfection of pASK/E3kbOp with pgfplacI to the cotransfection of pASK/E3kbOp with either control pET (compare the blue bars in group 6 to those in group 5) or pASK/E3kbOp alone (blue bar of group 4), it is clear that the lac repressor reduced β-gal gene expression. To further confirm that the repression of gene expression is caused by lacI/operator-formed complexes (via recruiting ND10 proteins), we added IPTG into the transfection or cotransfection system (Figure 5, yellow bars). IPTG can specifically bind to lacI and block the binding of lacI with the operator. As expected, the repression of gene expression can be partially but significantly rescued by adding IPTG (compare the yellow bar with the blue bar in group 6). This rescuing is a specific reaction of IPTG to the lac repressor because IPTG does not affect gene expression in other groups (groups 1–5 of Figure 5). In summary, we utilized the lacO/GFP-lac repressor system to detected the effects of ND10 on foreign gene expression and found that ND10 is a nuclear structure that represses gene expression.

## Discussion

The initial finding that DNA viruses from several families are deposited at specific nuclear domains and start their transcription and replication at these nuclear sites [49] suggested two contrasting hypotheses to explain this deposition. That ND10 might be the viral transcription cellular initiation site is most easily explained by assuming that there is some selective advantage for the virus that is provided by localizing at sites of high concentrations of potential transcription factors that may be recruited to the site in a combinatorial fashion. On the other hand, a nuclear defense mechanism might explain why viral genomes are deposited at sites of high concentrations of interferon-upregulated transcriptional repressors, as ND10 is also known to be a site of repression of viral transcription (for review, see references [12,22,49,50]. Neither hypothesis can be directly substantiated; both are based on the observed increase in the local concentration of proteins serving the virus for transcription or for repressing such transcription. Transcription factors or repressors apparently diffuse throughout the nucleus freely, and the advantage of such factors being in a bound state is difficult to rationalize. Viruses that do not reach ND10 should at least replicate moderately, given the availability of diffuse proteins recognizably concentrated at ND10. However, replication start sites appear to be associated with ND10.

Since the signal from a single viral genome and the associated proteins would be too low for visualization by immunofluorescence, we used a method that seemed to provide a comprehensive visualization of foreign DNA/protein complexes. The repetitive nature of the lacO repeats bound by the GFP-lac repressor enabled facile microscopic visualization and consequently the creation of an assay for proteins attracted to foreign DNA/protein complexes. The HPV/GFP-fused E2 complex recruited ND10-associated proteins and was found to associate with PML. This association, presented as a model for foreign protein expression [37], might demonstrate a general concept for recognition of a foreign DNA/protein complex since neither the foreign DNA nor the foreign protein alone associates with the ND10 protein. We also found that no transcription units were necessary for the recruitment of PML or other ND10 proteins to the foreign DNA/protein complex whether integrated or introduced by transfection, and no cellular repeat DNA in its normal genomic position or after transfection (presumed to exist as a DNA/protein complex) recruited ND10-associated proteins. Thus, the recognition of a foreign DNA/protein complex by three interferon-upregulatable proteins may represent an innate defense mechanism.

Several ND10-associated proteins are recruited to the bacterial and viral DNA/protein complex. An analysis of cell lines lacking individual ND10-associated proteins, such as PML, Daxx, or SP100, showed that none of these proteins was required to recruit the others. Each of these proteins is considered a repressor [51-56], suggesting the presence of a redundant repressor activity.

Several lines of evidence suggest that rather than nucleating new ND10 or moving into ND10 sites, ND10 proteins are recruited to the site of foreign DNA/protein complexes. First, integrated operator sequences neither localized at ND10 nor colocalized with ND10 proteins (Figure 1). Second, neither ND10 nor genomic loci (here the integrated operator repeat) move substantially. Third, the operator/repressor complex perfectly colocalizes with ND10 proteins, even in the absence of ND10 as a structure, whereas cellular DNA/protein complexes do not colocalize (although they may appear localized beside ND10). Thus, the recognition and accumulation of ND10-associated proteins is specific for the foreign DNA/protein complex and has been observed for HSV-1 components in a different context before [57,58]. Since recruitment of several ND10 proteins can occur in the absence of PML (absence of ND10 as a structure), we assume that the proteins accumulate from the nucleoplasm.

The ND10 protein-containing “foreign” aggregates contain DNA, which is most likely viral since cellular DNA has not definitively been found inside ND10 [59]. Although we cannot exclude the possibility that ND10 forms in association with some genomic loci [60], our evidence argues against this, at least for repetitive DNA or concentrated multiple cellular sequences. These sequences should exist as DNA/protein complexes and are sufficiently large or repetitive to bind proteins in quantities that can be visualized by immunohistochemical means. No PML, Sp100, or Daxx was found there. We prefer not to designate the protein accumulations induced by foreign DNA/protein complexes as ND10 nor as PML nuclear bodies since these aggregates are a response to the formation of foreign DNA/protein complexes and can develop without PML. That the large integrated foreign DNA was able to recruit ND10 protein to an aggregate size similar to that of ND10 appears purely as a function of the space occupied by such DNA.

If the introduced DNA binds ND10-associated proteins such as the interferon-upregulated repressors PML, Daxx, and Sp100, the expression of the gene carried by the DNA may be suppressed by these proteins. Here, we were able to facilitate the transfection of DNA to ND10. The β-gal gene expression assay showed that the input gene associated with ND10 has a decreased expression (Figure 5). Therefore, the results implied that ND10 is a defensive site rather than a site favoring gene expression. However, several questions still remain unanswered: 1) Can the operator/repressor complex system be used to bring a whole infected viral genome to ND10 at the time of infection? 2) The lac repressor protein is large and contains several domains, but its DNA-binding domain is relatively small (from aa 1 to aa 62); then can this small DNA-binding domain be used to set up the operator/repressor system for the visualization of viral replication? 3) Obviously, transcripts mostly accumulate at ND10 when the gene is targeted to ND10, and transcripts mostly diffuse in the nucleus if the gene is not targeted to ND10. The latter will have more gene transcription efficiency, which suggests that ND10 inhibits gene expression. How does ND10 detain transcripts and repress gene expression? We will further investigate the interaction of ND10 and foreign genes to answer all of these questions.

## Materials and methods

### Generation of stably transfected cell lines and tissue culture

HEp-2 cells were transfected using the DOSPER transfection reagent (Roche), either with plasmid pcDNA3lacO, which contains 10 kb of repetitive lacO sequences (256 repeats) or 5 kb of repetitive lacO sequences (128 repeats), or with plasmid pcDNA3gfplacI, which expresses the GFP-lac repressor (see below). Geneticin (400 μg/ml) was added on the second day after transfection. Surviving cell clones were selected and maintained in DMEM with 400 μg/ml Geneticin. Fluorescent *in situ* hybridization (FISH) identified the two clones from the cells transfected with the lacO repeats: HEp-2-Op (either 5 kb = 128 repeats or 10 kb = 256 repeats). HEp-2 cells were transfected with the gfplacI-expressing plasmid and selected to produce the GFP-lac repressor fusion protein, resulting in cell line HEp-2gfplacI. These cells were maintained in DMEM supplemented with 10% fetal calf serum (FCS), 1% penicillin/streptomycin, and 400 μg/ml Geneticin. Transfections and infections were performed in Geneticin-free medium. The cell lines MEF, PML−/−, and Daxx−/− have been described previously (61). SP100−/− 293 cells were selected from 293-T cells that do not produce SP100 (60). Vero (ATCC CCL-81), human fibroblast (ATCC 55-X), and HEp-2 cells (ATCC, CCL-23) were maintained in DMEM supplemented with 10% FCS and 1% penicillin/streptomycin. For immunohistochemical staining and for *in situ* hybridization, cells were grown on round coverslips (Corning Incorporated, Corning, NY) in 24-well plates. Cell fusion was induced by incubating mixed cultures of HEp-2-Op and HEp-2gfplacI cells, grown to over 80% confluency, in 50% PEG-1000 (Sigma) for 1 min. The cells were then washed several times with medium and incubated overnight at 37°C before fixation.

### Antibodies

ND10-associated proteins were visualized using the following primary antibodies: rabbit serum R14 produced against the N-terminal half of PML protein [11]; monoclonal antibody (mAb) 5.14 against human Daxx [61]; rabbit antibodies against murine Daxx (Santa Cruz Biotechnology, Inc., Santa Cruz, CA); mAb 1150 against human SP100 [62]; rabbit antibody against SUMO 1 (obtained from R M Tanguay, Quebec, Canada) [63]; and mouse antibody against ATRX (sc-15048, Santa Cruz, CA). HP1 was detected with a mAb obtained from F Rauscher [64] and mAb 138 labels NDP55 [65]. The mAb against IE1/2 (MAB810) was purchased from Sigma (Saint Louis, MO).

### HSV-1 amplicons

Amplicons were generated from plasmids containing the HSV-1 origin of replication [66] and packaging sequences (“a” sequences) [10,67,68]. To generate an HSV-1 amplicon, which contains lacO repeats, pASK/E containing HSV-1 ori-S and packaging sequences [10] was used to insert 10 kb operator DNA lacO repeats to make plasmid pASK/E-Op. pASK/E-Op was later transfected into Vero cells and superinfected with HSV-1 to make the amplicons [68]. Amplicon purification was performed according to the protocol that has been described previously [10].

### Plasmids and molecular cloning

placO, containing 256 or 128 lacO repeats, was derived from pSV2-dhfr8.32 (kindly provided by A Belmont) [33] by digestion with PvuII/BamHI and re-ligation, thus removing the SV40 origin and the dhfr sequence. pcDNA3gfplacI was generated by inserting the gfplacI sequence into pcDNA3 and was used to generate cell line HEp-2gfplacI. The gfplacI gene was obtained by PCR using p3’ssdimer-gfplacI (provided by A Belmont) [33] as the template and using the primers 5’CGAAAGCTTCGATGGTGAGCAAGGGCGAGG-3’ and 5’-TCAAACCTTCCTCTT CTTCTTAGG-3’. Plasmid pcDNA3lacO, containing 10 kb (or 5 kb) of repetitive lacO sequences (256 or 128 repeats), was used for transfection in order to generate the HEp-2-Op cell lines.

Plasmid pgfpE2, expressing HPV11 origin-binding protein fused with GFP (gfpE2), was provided by Dr. LT Chow [69]. A plasmid containing 128 repeats of HPV11 origin, pHPVori128, was cloned according to the method described above (used for making lacO repeats).

Plasmid pTP18 [70], which contains a complete U2 snRNA repeat cloned into pUC18, was used both as a probe to detect endogenous U2 genes and for transfecting U2 genes [38,71]. Plasmid HSE-LUC containing the promoter for heat shock protein 70 was obtained from Dr. R Voellmy (Miami, FL) [72] and transfected to test for additional cellular DNA, where binding to cellular proteins (heat shock factor) can be induced by 1 h exposure to 42°C, 16 h post-transfection.

### Immunocytochemistry and fluorescent *in situ* hybridization

Immunostaining was performed on cells grown on coverslips after fixation with 1% paraformaldehyde (10 min, room temperature) and permeabilization in 0.2% Triton (20 min on ice) by sequential incubation with primary and Texas Red- or FITC-labeled secondary antibodies (Vector Laboratories, Burlingame, CA) for 30 min each (all solutions in PBS). For simultaneous detection of ND10 and specific DNA or RNA sequences, cells were first immunostained for ND10 proteins. Cells were then treated for 1 h at 37°C with RNase-free DNase I (Boehringer; 200 U/ml in PBS containing 25 mM MgCl2) for the detection of RNA or with RNase (Boehringer, 100 ug/ml in PBS) for the detection of DNA. After refixation in 4% paraformaldehyde (10 min at room temperature), samples were equilibrated in 2X SSC, dehydrated in ethanol (70%, 80%, and 100% ethanol for 3 min each at −20°C), air-dried, and incubated overnight at 37°C with the hybridization solution. For DNA detection, the probe and the cells were simultaneously heated at 94°C for 4 min to denature DNA. To detect RNA, only the probe DNA was denatured (at 94°C for 5 min). After hybridization, samples were washed at 37°C with 55% formamide in 2X SSC (twice for 15 min each), 2X SSC (10 min), and 0.25X SSC (2X 5 min). Hybridized probes were labeled with FITC-avidin (Vector Laboratories; 1:500 in 4X SSC plus 0.5% BSA) and signals were amplified using biotinylated anti-avidin (Vector Laboratories, 1:250), followed by another round of FITC-avidin staining. Finally, cells were equilibrated in PBS, stained for DNA with Hoechst 33258 (0.5 ng/ml), and mounted in Fluoromount-G slide mounting medium (Fisher Scientific).

### Probe preparation

The following plasmids labeled with biotin-11-dUTP by nick-translation were used as probes: pRSVZ to detect lacZ RNA [30]; placO to detect lac operator repeats; and pHPVori128 to examine HPV origin DNA. The plasmid pTP18 served as a probe to visualize cellular U2 snRNA genes. In contrast, to detect transfected U2 genes (by transfection with pTP18) without labeling endogenous genes, only the vector sequences of pTP18 were hybridized, using pUC18 as probe. Collagen Iα1 and β-actin genes [41] and the non-coding mid-satellite sequence D1Z2 [39] were detected as described. The DNase concentration for nick-translation was adjusted to yield probe DNA 200 – 500 bp in length. Probe DNA was dissolved at 10 ng/ul in Hybrisol VII (Oncor, Inc., Gaithersburg, MD) containing 100 ng/ul salmon sperm DNA (Gibco BRL), 1 ug/ul yeast tRNA (Sigma), and 0.5 ug/ul cot1 DNA (Gibco BRL).

### Beta*-*galactosidase assay

Detection of the activity of β-galactosidase was carried out using GAL-1KT (β –Galactosidase Reporter Gene Activity Assay; Sigma-Aldrich), following the manufacturer’s protocol. Twenty-four hours after transfection of the plasmids (as indicated in Figure 5), the cells were washed three times with PBS and treated with lysis buffer (provided with the kit). Lysates of cells was collected in microcentrifuge tubes, and cell debris was removed by centrifugation. A sample with only lysis buffer was used to adjust the background for the optical density (OD) reader. Assay buffer was added to each sample and mixed by pipetting. The samples were then incubated for 30 minutes at 37°C. The reaction was terminated by a stop solution in order to proceed to the OD reading (420 nm in a multi-mode microplate reader; Synergy HT, BioTek). For the assays involving isopropyl-thio-b-D-galactopyranoside (IPTG), a final concentration of 0.4 mM was used. IPTG was added to the supplemented medium and left overnight. Each experiment was performed in triplicate, from which the average value was obtained.

### Confocal microscopy

Cells were examined at 100x magnification with a Leica TCS SPII confocal laser scanning system equipped with a water-cooled argon-krypton laser. Two channels (495 and 590 nm) were recorded either simultaneously or sequentially. Prior to data acquisition, power and integration were adjusted to minimize bleed-through between the green and far-red channels. Digital images obtained were cropped and adjusted for contrast with Photoshop. To balance the signal strength, we used Leica’s image-enhancement software to scan the image and separate the signal from its background. Because of the variability of cells in any culture in terms of size and shape, we selected the most typical cells that had been photographed, and they are presented here at high magnification.

## Competing interest

The authors declare that they have no competing interests.

## Authors’ contributions

YLM performed constructions of plasmids, immunofluorescent assays, beta*-*Galactosidase Assay and organized the data and participated in preparation of the manuscript. BBR performed cell culture and construction of several cell lines (HEp-2-op and HEp-2gfplacI) used in this paper. QT performed microscopy, analyzed the data and prepared the manuscript. All authors read and approved the final manuscript.
